# Novel Technologies for Improved Treatment Outcome and Patient Safety in Cancer Radiotherapy

**DOI:** 10.1155/2016/3016454

**Published:** 2016-03-01

**Authors:** Jun Deng, Yuanming Feng, Charlie Ma, Fang-Fang Yin

**Affiliations:** ^1^Department of Therapeutic Radiology, Yale University, New Haven, CT 06510, USA; ^2^Department of Biomedical Engineering, Tianjin University, Tianjin 300072, China; ^3^Department of Radiation Oncology, Fox Chase Cancer Center, Philadelphia, PA 19111, USA; ^4^Department of Radiation Oncology, Duke University, Durham, NC 27710, USA

Cancer radiotherapy has been largely driven by technological development in the past thirty years, resulting in improved local tumor control for a variety of lesion sites [1]. Yet the paradigm has shifted more recently toward a patient-centered healthcare model and value-based medicine where success is measured by improved treatment outcome, enhanced patient safety, and increased patient satisfaction [2]. Clinically, local tumor control should be optimized simultaneously with treatment outcome and patient safety in order to maximize the therapeutic ratio while maintaining a high standard of patient care. This special issue aims to present some of the novel technological developments in recent years and discuss their potentials in improving treatment outcome and patient safety in the radiotherapeutic management of cancers worldwide.

The research work entitled “An IMRT/VMAT Technique for Non-Small Cell Lung Cancer” investigated a novel approach in treating non-small cell lung cancer (NSCLC) with radiation, that is, a hybrid intensity-modulated radiotherapy (IMRT) and volumetric modulated arc therapy (VMAT) technique, in order to achieve the delivery efficiency from VMAT and the critical structure sparing with IMRT. The proposed hybrid approach involved an initial IMRT plan delivering the first half of the total prescription dose, followed by a VMAT plan delivering the second half of the total dose, which was optimized based on the initially optimized IMRT plan. With the dosimetric evaluation of 15 NSCLC patients, the hybrid technique has been shown to reduce *V*_5_, *V*_10_, *V*_30_, and the mean lung dose (MLD) of normal lungs compared with VMAT and better protect the organs-at-risk (OARs) with fewer monitor units (MUs) at the cost of slightly higher dosimetric indexes as compared to IMRT. The proposed hybrid approach demonstrated its promise in improving the treatment outcome and reducing normal tissue toxicity in the radiotherapy of lung cancers, which is one of the most deadly cancers in the modern era.

The clinical study entitled “Volumetric Modulated Arc Therapy of the Pelvic Lymph Nodes to the Aortic Bifurcation in Higher Risk Prostate Cancer: Early Toxicity Outcomes” addressed a controversial issue in the clinic, the treatment of pelvic lymph nodes (PLNs) in higher risk prostate carcinoma, by evaluating the early toxicity profile for this cohort of patients treated with VMAT. In this study, 113 patients treated with VMAT were enrolled with a median follow-up of 14 months. Acute and late genitourinary (GU) and gastrointestinal (GI) toxicities were documented as per the Radiation Therapy Oncology Group (RTOG) guidelines. Their study indicated that VMAT can be utilized efficaciously in a variety of indications to manage carcinoma of the prostate, especially in the high risk prostate cancer where pelvic lymph node volumes can be included up to the aortic bifurcation. This study clearly demonstrated that VMAT was a favorable treatment option in both the definitive and salvage settings in terms of reducing acute toxicity in GU and GI, which can lead to a better quality of life for patients with higher risk prostate cancer.

Another clinical investigation entitled “Replanning Criteria and Timing Definition for Parotid Protection-Based Adaptive Radiation Therapy in Nasopharyngeal Carcinoma” explored a clinically relevant and important issue, that is, the criteria and timing points of replanning for adaptive radiation therapy of nasopharyngeal carcinoma (NPC). Based on 50 NPC patients who were treated with helical tomotherapy, they evaluated the changes of volumetric and dosimetric indexes (*D*_mean_, *V*_1_, and *D*_50_) of the parotid glands at various fractional points. Their study indicated that initial parotid volume, initial parotid *D*_mean_, and weight loss rate were the most significant indicators for the parotid protection-based replanning in the adaptive radiotherapy of NPC. They further suggested three cutoff values which could be used to predict the timing for replanning in order to better protect the parotid glands during radiotherapy. This interesting clinical study emphasized the importance of patient safety and normal tissue sparing in order to improve the treatment outcome and quality of life of cancer patients.

Another research paper entitled “3D-2D Deformable Image Registration Using Feature-Based Nonuniform Meshes” proposed a novel feature-based nonuniform meshing method to efficiently register 3D images such as CTs with a small portion of 2D projection images of cone-beam CT in image-guided radiotherapy of cancers. In this work, they evaluated and compared the new 3D-2D deformable image registration (DIR) algorithm with other 3D-2D DIR methods in terms of image visualization and quantitative evaluations with two XCAT phantoms and five head and neck cancer patients. Compared with the traditional voxel-based DIR method, the proposed algorithm demonstrated faster computational speed and higher quality of reconstructed images. This technique could be used for accurate deformable image registration and fast adaptive radiotherapy replanning for better tumor control.

Finally, the review article entitled “Combining Whole-Brain Radiotherapy with Gefitinib/Erlotinib for Brain Metastases from Non-Small-Cell Lung Cancer: A Meta-Analysis” conducted systematic review and meta-analysis on the efficacy and safety of whole-brain radiotherapy (WBRT) combined with gefitinib/erlotinib for treatment of brain metastases (BM) from non-small cell lung cancer (NSCLC). Through an extensive literature search, a total of 7 case-controlled and randomized controlled trials involving 622 patients were included in this retrospective study. Statistical analyses indicated that WBRT plus gefitinib/erlotinib can significantly improve the response rate, the remission rate of the central nervous system, the disease control rate, the overall survival, and the one-year survival rate in patients with BM from NSCLC, as compared to WBRT alone or WBRT plus chemotherapy.

In general, this special issue presents a snapshot of what is being actively pursued currently in cancer radiotherapy. In addition to the technological developments featured in this special issue, there have been numerous exciting developments on a variety of topics, such as advanced imaging for treatment response assessment, advanced tumor tracking and monitoring, online quality assurance of treatment delivery, advanced algorithms for real-time monitoring of patient safety, biologically guided radiation therapy, and electronic health record data mining for more efficient radiotherapy. The successful implementation of these novel technologies into routine clinical workflow will undoubtedly improve patient safety and treatment outcome in the radiotherapeutic management of cancers and usher in a new paradigm for a more personalized radiation therapy.

*Jun Deng* *Yuanming Feng* *Charlie Ma* *Fang-Fang Yin*
